# Deep Ensemble Learning with Atrous Spatial Pyramid Networks for Protein Secondary Structure Prediction

**DOI:** 10.3390/biom12060774

**Published:** 2022-06-02

**Authors:** Yuzhi Guo, Jiaxiang Wu, Hehuan Ma, Sheng Wang, Junzhou Huang

**Affiliations:** 1 Department of Computer Science and Engineering, University of Texas at Arlington, Arlington, TX 76019, USA; yuzhi.guo@mavs.uta.edu (Y.G.); hehuan.ma@mavs.uta.edu (H.M.); sheng.wang@mavs.uta.edu (S.W.); 2 AI Lab, Tencent, Shenzhen 508929, China; jonathanwu@tencent.com

**Keywords:** deep learning, protein secondary structure prediction, learning representation, computational biology

## Abstract

The secondary structure of proteins is significant for studying the three-dimensional structure and functions of proteins. Several models from image understanding and natural language modeling have been successfully adapted in the protein sequence study area, such as Long Short-term Memory (LSTM) network and Convolutional Neural Network (CNN). Recently, Gated Convolutional Neural Network (GCNN) has been proposed for natural language processing. It has achieved high levels of sentence scoring, as well as reduced the latency. Conditionally Parameterized Convolution (CondConv) is another novel study which has gained great success in the image processing area. Compared with vanilla CNN, CondConv uses extra sample-dependant modules to conditionally adjust the convolutional network. In this paper, we propose a novel Conditionally Parameterized Convolutional network (CondGCNN) which utilizes the power of both CondConv and GCNN. CondGCNN leverages an ensemble encoder to combine the capabilities of both LSTM and CondGCNN to encode protein sequences by better capturing protein sequential features. In addition, we explore the similarity between the secondary structure prediction problem and the image segmentation problem, and propose an ASP network (Atrous Spatial Pyramid Pooling (ASPP) based network) to capture fine boundary details in secondary structure. Extensive experiments show that the proposed method can achieve higher performance on protein secondary structure prediction task than existing methods on CB513, Casp11, CASP12, CASP13, and CASP14 datasets. We also conducted ablation studies over each component to verify the effectiveness. Our method is expected to be useful for any protein related prediction tasks, which is not limited to protein secondary structure prediction.

## 1. Introduction

The three-dimensional structure of proteins is significant in the studies of proteins since the specific shape of a protein determines its function [1]. The protein may become denatured and not function as expected if its tertiary structure is altered due to mutations in the amino acid structure. Proteins are chains of amino acids linked by peptide bonds. However, predicting the three-dimensional structure of proteins from amino acid predictions is a challenging task [2]. Protein secondary structure prediction is an important part of this task [3,4,5,6,7,8].

Protein secondary structure prediction takes primary sequences as the input, which are the amino acid sequences of proteins, to predict the secondary structure type of each amino acid. Q3 accuracy is often used to evaluate the secondary structure: helix (H), strand (E), and coil (C), where the former two are regular secondary structure states and the last one is the irregular type [9]. Another definition of secondary structure is extends the three general states into eight fine-grained classes [10]: 3${}_{10}$ helix (G), α-helix (H), π-helix (I), β-stand (E), β-bridge (B), β-turn (T), high curvature loop (S), and others (L). Recently, the studies of secondary structure prediction has focused more on the prediction of 8-state secondary structure (Q8) instead of the 3-state(Q3) prediction. The reason is that a chain of 8-state secondary structure naturally contains more structural information for a variety of research and applications [11,12].

The methods of secondary structure prediction can be divided into template-based and template-free. Although template-based methods usually achieve better results [13], it does not work well on proteins with very low similarity with those sequences with known structures in the PDB [14] library. However, these proteins can be considered as newly discovered proteins, which is more like a real-world scenario. While template-free methods have used several traditional machine-learning models such as probabilistic graphical models [15,16], hidden Markov models [17,18], and Support Vector Machines (SVM) [19,20].

In recent years, deep learning techniques have been widely used in protein secondary structure prediction task, and achieved remarkable results compared with traditional machine learning methods. Several work explore the power of feed-forward back-propagation neural network (BPNN) with traditional machine learning models for protein secondary structure prediction [21,22,23], e.g., ref. [21] integrate BPNN with Bayesian segmentation, ref. [22] designs an architecture of protein secondary structure prediction by combining BPNN with SVM, etc. Later, DNSS [24] first proposes deep learning based secondary structure prediction method [7], which utilizes a deep belief network [25] based on restricted Boltzmann machine (RBM) [26]. Recently, more studies seek to involve more additional features such as position-specific scoring matrix (PSSM) features to further improve prediction performance [4].

In addition, sequence based models such as Recurrent Neural Network (RNN) encoder are used on protein sequence to predict protein secondary structures [27] and the first application of LSTM-BRNN to secondary structure prediction can be found in [28]; and one-dimensional Convolutional Neural Network (1d-CNN) based encoder method has also been used on such task and obtained some achievements [29]. Moreover, some studies tackle this problem by combining the superiority of different networks, e.g., DeepACLSTM [30] uses CNN to capture the local feature and bidirectional Long Short-term Memory Network (bLSTM) to obtain the long-distance dependency information. In such a manner, DeepACLSTM is able to obtain better amino acid sequence expression and achieve better prediction performance. Other methods that equipped with DeepCNN network [29] or ResNet [31] structure can also capture the long-distance dependency information from the sequence, e.g., CBRNN [11] combines the CNN-based and RNN-based networks.

**Figure 1:**
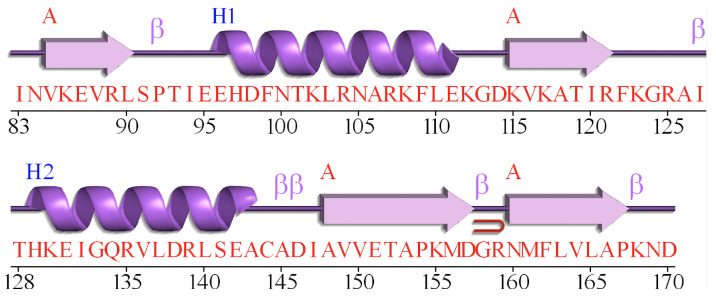
Protein 1TIG: Different symbols in purple represent different secondary structures, and red characters represent amino acid sequence. This figure is generated by PDBsum [37].

Although amino acid sequence encoders based deep learning methods have achieved great success, the relationship among the secondary structures of proteins is rarely studied. DeepCNF [29] method employs Conditional random field (CRF) as the output layer to learn the interdependency among adjacent secondary structure labels. However, it does not take the specific characteristic of protein secondary structure into consideration, and the improvement of Q8 accuracy is limited. Figure 1 illustrates the secondary structure and the amino acid sequence of protein 1TIG [14] from CB513 dataset, which is generated by PDBsum [32]. We can observe that adjacent strings of amino acids generally contain the same secondary structure. The reason of such well-regulated feature might be caused by the characteristics of the protein secondary structure. Consequently, this problem is quite similar to the image semantic segmentation (ISS) [33] problem. However, there exists two differences: (1) The input data of protein secondary structure task is one-dimensional sequences, while the images contains two dimensions. (2) For ISS, a pooling layer is widely used since the pooling of the adjacent pixels can effectively reduce the size of the input image [33,34,35,36]. Such implementation can reduce the network parameters while retain most of the image information. However, the amino acid information at each position is crucial for protein sequence, the pooling layer is not adoptable for the amino acid sequence.

Additionally, even various encoders have been proposed to address ISS, e.g., FastFCN [35], GSCNN [34], and all versions after Deeplab v2 [33,36], the Atrous Spatial Pyramid Pooling (ASPP) Network Structure [33,38] followed by the encoder still plays an important role to identify the boundaries of objects in the image.

Recently, CNN-based encoding models have obtained great success on both image and language processing tasks. Gated Convolutional networks (GCNN) [39] employs a CNN-based gating mechanism at the channel level to help the language modeling. Conditionally Parameterized Convolution (CondConv) [40] uses extra sample-dependant modules to conditionally adjust the convolutional network, which has obtained remarkable improvement over the image processing tasks. In this paper, we present a novel protein sequence encoder, Conditionally Parameterized Gated Convolutional network (CondGCNN), which not only exploits a gating mechanism at the channel level, but also establishes a sample-dependent attention mechanism.

Inspired by previous work about the protein secondary structure prediction task and the ISS, we propose a protein ensemble learning method with ASP networks, which contains an ensemble amino acid sequence encoder and Atrous Spatial Pyramid Networks. Since CNN-based methods have obtained remarkable performance in language modeling and image processing tasks, and lstm-based methods are important for protein prediction [27,30], our amino acid sequence encoder has utilized both CondGCNN model (a new encoder we proposed) and bLSTM model. Besides, the ASP Network (optimized ASPP network for our problem) is added following the encoder.

The technical contributions of proposed method can be summarized as: (1) The work is the first to tackle protein secondary structure prediction task with image segmentation processing, which utilizes the predominance of those models applied in the segmentation area to tackle secondary structure prediction problem, e.g., employ ASPP network (optimized as ASP network in our method) to capture fine edge details in secondary structure labels. (2) We are the first to apply CondConv network on sequence processing problems, as well as embed it in the GCNN to form a novel amino acid sequence encoder. In specific, a gating mechanism is equipped at the model channel level and a sample-dependent attention is employed at the input level. (3) We construct an ensemble encoder with cnn-based and lstm-based networks, which has acquired more diverse information from amino acid sequences. (4) Through a set of extensive ablation studies, the significance of different components of the method, including architecture, features, and results, are carefully analyzed. Based on our conference version of the paper [41], we design more experiments to verify the effect of our ASP module on the boundary residues prediction and verify the effectiveness of our framework on more datasets.

The rest of the paper is organized as follows. In Section 2, we describe the framework and modules used in our method in detail. Section 3 gives the experimental results to demonstrate the advantage of our method. Finally, in Section 4, we conclude our paper and discuss its prospect.

## 2. Materials and Methods

In this section, we describe the details of our method. First, we introduce the datasets and the input feature. Second, we give a brief introduction to the framework of our method. Then, we propose an ensemble encoder composed of two modules: CondGCNN module and LSTM module. Next, we introduce the Atrous Spatial Pyramid Network as the secondary structure generator (generation module) of our method and explain the improvements to the traditional ASPP network and the output layer of the secondary structure prediction task. Finally, we illustrate the network structure of the comparative experiment.

### 2.1. Datasets

We use CullPDB [42] publicly available dataset for training and validation. 501 proteins in CullPDB dataset are randomly sampled for validation, then the remaining proteins are used for training. Proteins in the CullPDB dataset share no more than 25% sequence identity [29] with our other datasets (CB513, CASP11, CASP12, CASP13, CASP14) for testing. CB513 [5] dataset is commonly used for testing and comparing the performance of the protein secondary structure prediction methods [29,30,43]. The dataset contains 513 proteins and is obtained from [5]. As the critical assessment of protein structure prediction since 1994, the CASP datasets have been also widely used in the protein studying community [44]. The 85 proteins in CASP11, 40 proteins in CASP12, 10 proteins in CASP13, and 15 proteins in CASP14 are used as our CASP datasets (http://predictioncenter.org, accessed on 3 March 2022). Note that we only use the template-free proteins for CASP13 and CASP14, which are obtained from the official websites http://predictioncenter.org/casp13/domains_summary.cgi and http://predictioncenter.org/casp14/domains_summary.cgi (accessed on 3 March 2022). The secondary structure labels for datasets are generated by DSSP [10]. More details of the Q8 secondary structures in these datasets are listed in Table 1.

Furthermore, we explore larger test datasets to thoroughly evaluate the performance of our method. SPOT-1D [45] announces a benchmark dataset which contains 10,200 proteins for training; 1000 proteins for validation; and two independent test sets test2016 and test2018 with 1213 and 250 proteins, respectively. More details about the SPOD-1D benchmark can be found at [45]. We conducted extensive experiments on the provided datasets follow our settings. In specific, we take the protein and PSSM sequences as the input, and the protein 8-state secondary structure as the labels to form a SPOT-1D benchmark dataset. Extensive experiments of ours and baseline methods are conducted over SPOT-1D benchmark dataset to compare the prediction performance.

### 2.2. Input Feature

**Figure 2:**
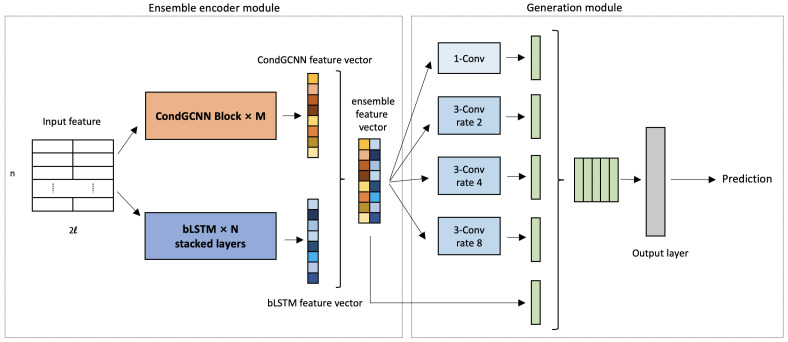
Our ensemble learning with ASP networks framework contains ensemble encoder module and generation module. For ensemble encoder, we use several CondGCNN blocks and bLSTM layers in the networks; for generation module, a modified ASPP is applied in the module.

Our input feature consists of two parts: sequence one-hot vectors and position-specific scoring matrix (PSSM). Each amino acid in the protein sequence is represented by a one-hot vector with length as 21, which refers to 20 kinds of amino acids plus one unknown amino acid. PSSM represents the distribution of amino acid types on each position in the protein sequence [46]. Following the same procedure in [5,41,47], we get the PSSM matrix by searching Uniref50 database [48], and concatenate it with the one-hot vectors. As shown in Figure 2, the input feature size is n×2l where l=21 and *n* is the length of the protein sequence.

### 2.3. Deep Learning Framework Overview

The framework of our method is constructed by the ensemble encoder module and the generation module. We will introduce each component in this section. The overall workflow is illustrated in Figure 2. First, the input sequence features are fed into the CondGCNN and the LSTM modules respectively. Next, the outputs of two network are concatenated as the feature vectors to feed into the generation module. Last, the loss is calculated by the output prediction and secondary structure label, and back-propagated to the networks for parameters adjustment.

### 2.4. Ensemble Encoder

The ensemble encoder module includes one CondGCNN module and one LSTM module. The CondGCNN module contains M × Conditionally Parameterized Gated Convolutional blocks, while the LSTM module is constituted by N stacked bLSTM. These two modules generate output feature vectors respectively.

#### 2.4.1. CondGCNN Module

**Figure 3:**
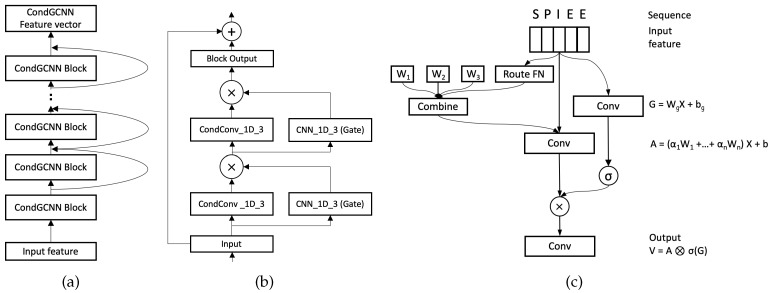
(**a**) CondGCNN encoder contains 32 blocks to get the feature vectors. (**b**) Each block contains 2 layers of Conditionally Parameterized Gated Convolutional network. The input vector of *Input* block is added to the output vector of the *Block Output*, the combination then input to the next block. (**c**) One layer of CondGCNN contains two parallel convolutional layers, one is the conditionally convolutional layer (A) and the other one is the gated layer (G). The output V is obtained by the element-wise production of A and σ(G).

Figure 3a,b demonstrate our CondGCNN blocks. 32 CondGCNN blocks are used to get the feature vectors in the CondGCNN encoder. Each CondGCNN block contains two layers of Conditionally Parameterized Gated Convolutional network. We build our CondGCNN layers follows [39,40]. Figure 3c illustrates the architecture of each CondGCNN layer. A protein sequence is represented by a n×2l vector, where *n* is the length of the protein sequence and *l* is the number of amino acid types. The details about the input features are discussed in Section 3.1. For each CondGCNN layer, we set up two CNN_1D_3 networks, one is used for gating, and the other one is a one-dimensional Conditionally Parameterized Convolutional network. We calculate the output vector of the CondGCNN layer follows:

(1) $$V_{h}\left(X\right)=\left(X*W_{cond}+b\right)⊗\sigma \left(X*W_{g}+b_{g}\right),$$

where $W_{cond}$ and *b* are the parameters of the CondConv network, $W_{g}$ and $b_{g}$ are the parameters of the gated convolutional network, σ is the Sigmoid function, and ⊗ refers to the element-wise product between vectors. More details of the GCNN network can be found in [39]. Specifically, we parameterize the convolutional kernels in CondConv by:

(2) $$W_{cond}=\alpha_{1}\cdot W_{1}+\alpha_{2}\cdot W_{2}+\cdots +\alpha_{n}\cdot W_{n},$$

where each $\alpha_{i}=r_{i}\left(X\right)$ means an example-dependent scalar weight computed using a routing function with learned parameters, and *n* stands for the number of experts. The routing function is able to meaningfully differentiate between the input examples. CondConv [40] computes the example-dependent rounting weights $\alpha_{i}$ from the layer input in three steps: global average pooling, fully-connected layer, and Sigmoid activation.

(3) r(X)=Sigmoid(GlobalAveragePool(X)R)

where *R* is a matrix of the routing weights mapping the pooled inputs to *n* expert weights.

Overall, our CondGCNN encoding module utilizes the predominance of both CondConv and GCNN, which not only provides a gating mechanism at the channel level, but also implements an attention mechanism in a sample-dependant fashion.

#### 2.4.2. LSTM Module

Some studies about language modeling with the GCNN [39] claim that unlimited contextual information is unnecessary for language models, and GCNN is proved to be able to represent enough contextual information in practice. However, in the area of protein study, several works have proved that capturing the long contextual information (relation from the first atom to the last one) is necessary. Therefore, RNN-based approaches are crucial for protein studies [11,30]. Recurrent neural networks (RNNs) have been applied in sequence-process modeling and achieved remarkable performance, however the gradient vector may fluctuate exponentially over long input sequences during training process. Therefore, LSTM network introduce gate structure to handle the problem [49]. LSTM implements three gates: input gate $i_{t}$, forget gate $f_{t}$ and output gate $o_{t}$ and a memory cell $c_{t}$, where *t* is the time step. Formally, one unit of LSTM can be computed as:

(4) $$i_{t}=\sigma \left(X_{t}W_{i}+h_{t-1}W_{i}+b_{i}\right)$$

(5) $$f_{t}=\sigma \left(X_{t}W_{f}+h_{t-1}W_{f}+b_{f}\right)$$

(6) $$o_{t}=\sigma \left(X_{t}W_{o}+h_{t-1}W_{o}+b_{o}\right)$$

(7) $$g_{t}=tanh\left(X_{t}W_{g}+h_{t-1}W_{g}+b_{g}\right)$$

(8) $$c_{t}=\sigma \left(f_{t}⊗c_{t-1}+i_{t}⊗g_{t}\right)$$

(9) $$h_{t}=o_{t}⊗tanh\left(c_{t}\right)$$

where σ is the Sigmoid function; *W* and *b* represent the corresponding weight matrix and bias term; ⊗ is the element-wise multiplication. In Equation (9), the $h_{t}$ is the hidden vector, which is computed by the current input $x_{t}$ and the previous $h_{t-1}$, where *t* is the current time step.

In this fashion, our proposed method implements two stacked bLSTM layers with a hidden 512 within the LSTM module to capture more long-distance interdependencies of amino-acid residues.

A bLSTM neural network consists of two LSTM neural networks in parallel, one of them runs on the input features and the other one runs on the reverse of the input features. The two corresponding output vectors are then concatenated as the feature vector for LSTM module. More details regarding stacked bLSTM network can be referred in [30,50].

### 2.5. ASP Generation Module

As shown in Figure 2, we feed the concatenated feature vector from Ensemble encoder into the generation module for the protein secondary structure prediction. The generation module contains the Atrous Spatial Pyramid Network and the output layer.

#### 2.5.1. Atrous Spatial Pyramid Network

**Figure 4:**
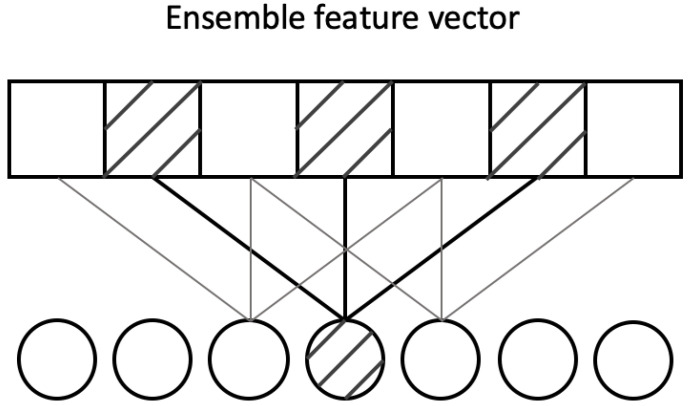
An example of Atrous one-dimensional convolutions (one layer) with dilation rate equal to 2: A 3 × 1 kernel with a dilation rate of 2 has the same field of view as a 5 × 1 kernel, which provide a wider field of view with the same computational cost.

As we have mentioned before, the secondary structure prediction task for proteins is similar to the semantic segmentation tasks for images. For ISS, the model needs to classify each pixel with one of the predetermined classes. Similarly, in protein secondary structure prediction, we need to classify eight secondary structures of amino acids for each position. In addition, the labels of protein secondary structure behave consistently for adjacent positions too. Our generation model is inspired by the ASPP network, which is widely used in image segmentation [33,34,35]. The ASPP network is proposed by Deeplab [33], which uses dilated convolutions with different rates instead of regular convolutions, as an attempt of classifying regions of an arbitrary scale. Specifically, the essence of ASPP network is the use of atrous convolutions, which originally developed for the undecimated wavelet transform efficient computation [51]. The algorithm is a powerful tool which allows us to compute responses of any deep convolutional layer and adjust filter’s view to capture information at any desirable resolution. It can be applied to a model has been trained, but can also be seamlessly embedded into training process. Considering one-dimensional signals in our task, the output feture map *y* on each location *i* of atrous convolution of a one-dimension input signal x[i] with a convolution filter *w* of length *K* is defined as follow:

(10) $$y\left[i\right]=\sum_{k=1}^{K}x\left[i+r\cdot k\right]w\left[k\right]$$

where the rate parameter *r* determines the stride with the input signal we sampled. We refer [33] for more details. Note that when r=1, it is a special case which is the standard convolution. Figure 4 demonstrates an example of one layer Atrous Spatial Pyramid network, as shown, the dilation rate of each Atrous (dilated) convolutional layer is set as 2, the rate of the normal Convolutions is 1.

However, ASPP based networks have rarely been applied to sequence problems, especially in the prediction of secondary structure of proteins. Down-sampling is a commonly used method to reduce the size of feature map in the image processing field, ASPP networks use two down-sampling mechanism: one is the convolution striding, and the other one is using pooling operations (max-pooling or average-pooling). ASPP sets the stride equal to 8 for each convolutional layer in the networks, and processes the image-level features via Global Average Pooling (GAP) [52].

In our work, protein sequences are usually short in length (around one hundred) and each position in the sequence is important, we set the convolution stride to 1 and concatenate the ensemble feature vector with the outputs from four convolutional layers in the networks directly in stead of a pooling layer. Since very high dilation rate is not needed for our task, we set (2, 4, 8) as the dilation rates.

#### 2.5.2. Output Layer

As shown in Figure 2, after the Atrous Spatial Pyramid network, we feed the result to a one dimension convolution with window size 1, to produce the final predicted secondary structure logits. Fully connection layer (FC) is widely used in LSTM-based secondary structure prediction methods. However in our task, since the Atrous Spatial Pyramid networks apply multiplication on the channel of the feature vector, the network would contain too many parameters if we implement Fully connected layers as the output layer, which makes the entire model hard to train. Thus, we replace the output layers with a one-dimensional convolutional layer. To prove the effectiveness of this change, we report the extensive experimental results in Section 3. The learning objective function is to minimize the cross-entropy loss function.

## 3. Results

In this section, we first introduce the experimental settings, such as the the applied neural network structures, hardware and software settings, boundary evaluation settings. Then we report the Q8 accuracy of two encoders respectively: lstm-based and cnn-based secondary structure prediction, as well as the improvement of existing methods by ASP network. Finally, by comparing with the state-of-the-art methods, we prove the superiority of our method.

### 3.1. Experiments Set Up

#### 3.1.1. Neural Network Structure and Learning Hyper-Parameters

In the CondGCNN module, we use 32 Conditionally Parameterized Gated Convolutional blocks. Each block contains two layers of CondGCNN with a window size 3 and a node size 64, the number of experts is 3. In the LSTM module, we use the two stacked layers bLSTM networks with hidden size equals to 512. In the ASP network, we utilize three parallel dilated convolutional layers with window size 3, node size 100, dilation rates = (2, 4, 8), and a parallel one-dimensional convolutional layers with window size equals to 1. We use a one-dimensional convolutional layer with window size to 1 and node size is equal to 100 as the output layer.

#### 3.1.2. Training Strategy

We use multi-step learning rate scheduler descent with [30,50] for epoch indices. The multiplicative factor of LR decay (learning rate) is 0.1. For optimizer, we use Adam [53] optimizer in our method. The initial LR for training is 0.001.

#### 3.1.3. Comparison Methods

To evaluate our method, we compare it with five following state-of-the-art methods: ICML2014, DeepCNF, MUFOLD-SS, CBRNN, and DeepACLSTM. Chosen either for their state-of-the-art performances or because they represent a class of prediction networks for secondary structure. ICML2014 [5] presents a method based on GSN (generative stochastic network) to globally train the deep generative model. We use the public dataset they provided, the CullPDB dataset containing 5926 Program database (PDB) files, and CB513 contains 513 proteins. DeepCNF (Deep Convolutional Neural Fields) [29] utilizes the power of CNN and Conditional Random Fields (CRF): five CNN layers are used to extract the sequence feature of proteins, and CRF is used as the output layer to catch the relationship between the predicted target. MUFOLD-SS [44] is a deep inception-inside-Inception (Deep3I) network architecture that extends deep inception networks through nested inception modules. Stacked inception modules can extract non-local residue interactions at different ranges. CBRNN [11] extracts the local context information of protein sequence by two-dimensional convolutional neural networks (2dCNNs), and long-distance information by bidirectional gated recurrent units (bGRUs) or bidirectional long short-term memory (bLSTM). DeepACLSTM [30] using 1-dimension CNN and 2-dimension CNN to extract the discriminational local interactions between amino-acid residues and bLSTM to capture long-distance interactions between amino-acid residues.

#### 3.1.4. Evaluation Metric

Following the [45,50] instruction, we use Q8 accuracy (higher is better) as the evaluation metric for the prediction task of secondary structure. We use the Train-Valid-Test split method to evaluate the performance.

#### 3.1.5. Boundary Evaluation Set Up

**Figure 5:**
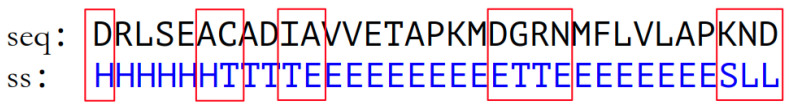
An example of boundary residue: the amino-acid residues in the red box. The first line is the amino acid sequence, and the second line is the secondary structure (ss) label sequence, which is the ground truth value of the secondary structure at the position of the corresponding residue.

In addition to reporting the experimental results compared with state-of-the-art methods, we also design an boundary evaluating criteria. Since the Atrous Spatial Pyramid Pooling (ASPP) networks was designed to help with identifying the boundary of objects in the image segmentation task [33,36], the ASP networks (a modified version of ASPP) in our model is aimed to identify the boundary of successive amino acids having the same secondary structure. In addition, prediction of boundary residues is always more challenging in secondary structure task in protein prediction, and we demonstrated this property in the subsection of extension experiments. We define a residue as a boundary residue if the secondary structure label of a residue is different from that of its adjacent residue (left adjacent or right adjacent). As shown in Figure 5, the amino-acid residues in the red box are boundary residues, and we will calculate the boundary Q8 accuracy for those part of residues to evaluate the boundary identifying ability of our model. The first line in the example is the amino-acid sequence, and the second line is the secondary structure (ss) label sequence, which is the ground truth value of the secondary structure at the position of the corresponding residue. We report the extensive experimental result of boundary residue in Section 3.4.

#### 3.1.6. Infrastructure and Software

Our model is implemented through Pytorch package. And our models is trained in a self-hosted 2-GPU server platform with Intel i7 6700K @ 4.00 GHz CPU, 64 Gigabytes RAM and two Nvidia GTX 2080Ti GPUs.

### 3.2. Ablation Study on Each Component

In this subsection, we report and analyse the various components of our framework contribute to final performance, including optimization and architectural choices. Unless stated otherwise, all experiments for the ablation studies follow the training strategy described in Section 3.1.4.

Table 2 shows the prediction results of Conv, CondConv, GCNN, and CondGCNN with different structures on CB513 dataset. First, we compare the results between the regular Convolutional network (Conv) and the Conditionally Parameterized Convolutional network (CondConv) on CB513 dataset to prove the effectiveness of the CondConv. We follow the settings of [29] to build a model with 5 layers of 1-dimdimension Convolutional networks, then apply the CondConv structure on the regular Convolutional networks directly. However, the improvement on accuracy is only 0.02. The reason is that when CondConv is applied, it will use more parameters to focus on distinguishing different samples compared with the traditional convolutional network since the attention mechanism works in a sample-dependant manner. This will lead to the overfitting problem. To overcome this disadvantage, we adjust the dropout rate and conduct extensive experiments with different number of experts. Furthermore, we report the prediction results of GatedCNN (GCNN) with different numbers of res-blocks on the protein secondary structure prediction task. As observed, the best result is 0.698 when using 32 GCNN blocks. Since we do not have quite a large training dataset, the over-fitting problem would be severe if the network is too deep. Hence, with the increase of blockes, the accuracy results of the validation and test sets are reduced significantly. Last, we compare our CondGCNN method with the above CNN-based methods, and the application of CondConv on the basis of GCNN can achieve 0.702 of Q8 accuracy on CB513 dataset.

**Figure 6:**
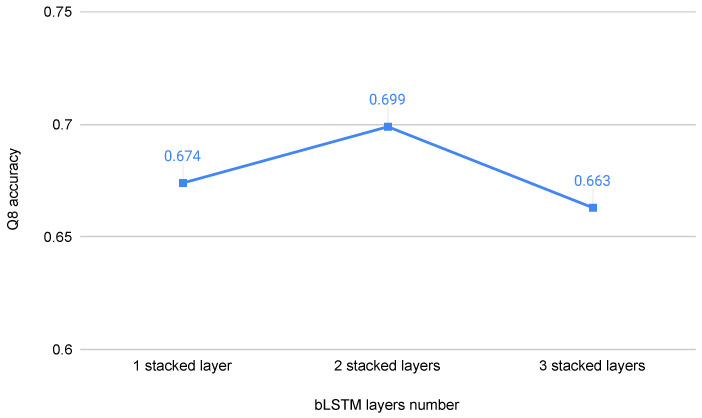
Q8 accuracy of bLSTM on cb513 dataset with different number of LSTM layers.

We report the experimental results of the bLSTM of different layer number, and the experimental settings are entirely in accordance with [27]. As shown in Figure 6, the prediction accuracy of two stacked layers bLSTM is higher than that of one layer and three layers bLSTM.

In order to prove the effectiveness of our Atrous Spatial Pyramid networks (ASP) module, we employ ASP module with LSTM method and DeepACLSTM method. We have noted that the performance is not as expected when directly insert the ASP module between the encoder and the output layer. The reason is that these two methods have used fully connected (FC) layer as the output layer, along with the augmented output of ASP network, leads to overfull parameters. The model is then too hard to train and easily to overfit. To reduce the overhead, we replace the output layer with a one-dimensional convolutional layer with a window size 1 and re-do the experiments. The extensive corresponding results are shown in Table 3 and Table 4. bLSTM-FC represents the original two stacked layers bLSTM networks structure [27], and ACLSTM-FC represents the DeepACLSTM network structure with FC as the output layer [30]. We use bLSTM-ASP-FC and ACLSTM-ASP-FC to indicate the methods that our ASP networks are inserted between the original encoders (bLSTM and DeepACLSTM) and FC layer. bLSTM-ASP-Conv1 and ACLSTM-ASP-Conv1 represent that FC layer is replaced by a 1d-cnn with window size 1 as the output layer after the ASP network. The results demonstrate that applying ASP directly to bLSTM and DeepACLSTM networks does not perform well for prediction. Nonetheless, after replacing the output layer with 1d-CNN, we promote the performance of LSTM method by 0.4% and DeepACLSTM method by 0.6%. The results prove that our ASP network can boost the existing state-of-the-art methods of protein secondary structure prediction. In addition, the hidden size (HS) of FC and the node size (NS) of ASP and Conv1 are also shown in Table 3 and Table 4.

### 3.3. The Results of Ensemble Learning with ASP

After conducting a series of experiments to prove the effectiveness of each component, we then combine them to build our network: Ensemble learning with Atrous Spatial Pyramid networks. To demonstrate the effectiveness of our model, we report the results of CB513, CASP11, CASP12, CASP13, CASP14 datasets to compare with several state-of-the-art methods.

Although we report extensive hyper-parameter search for each module, we perform the search space, as well as the parameters of our ensemble model with highest Q8 accuracy on the validation set. Table 5 shows the hyper-parameter space and best values for our Ensemble-ASP model. The “fc” represent the fully connected layer and the conv1 represent the convolutional layer with window size one.

Folowing the best hyper-parameter above, we report the overall experimental result. As shown in Table 6, the “Ensemble” represents our method without the ASP network, the “Ensemble-ASP” illustrates the result of Q8 accuracy after inserting the ASP network. Our method achieve more than 1% accuracy improvement over other state-of-the-art methods on CB513, CASP11, CASP13 and CASP14 datasets, and get around 0.7% higher on CASP12. The proposed model has not only utilized the power of CondGCNN and bLSTM, but also successfully applied the ASP network on protein secondary structure prediction task to obtain significant improvement.

### 3.4. Comparison Results on SPOT-1D Benchmark Dataset

In order to thoroughly evaluate the performance of our method in real-world application, we conduct comparison experiments using a much larger benchmark dataset, SPOT-1D, which contains a large test dataset with 1213 proteins. We follow the same experimental settings as our other experiments, where the protein sequences and PSSM features are used as input, and the protein 8-state secondary structure are used as the labels. We run all the comparison experiments follow the provided dataset splits, where the model is trained on the training dataset, and tested on two test sets (test2016 and test2018) with the model obtains best validation score. As shown in Table 7, our method achieves 0.5% and 0.6% improvement on test 2016 and test 2018, respectively.

### 3.5. Extension Experiments on Boundary Evaluation

In the above sections we report the overall results of applying Atrous Spatial Pyramid (ASP) networks to BLSTM, ACLSTM, and our Ensemble method. We define a residue as a boundary residue if the secondary structure label of the residue is different from that of its adjacent position (left adjacent or right adjacent). Here we only record the Q8 accuracy of boundary residues. Due to the small number of boundary residues in the whole amino acid sequence, we chose a relatively large test set CB513 to report the boundary validation results. As shown in Table 8, the Q8 accuracy of boundary residues is indeed much lower than the overall accuracy, and our ASP module can significant improve the performance of bLSTM, ACLSTM, and our Ensemble encoders in boundary residue prediction. Q8 acc is used as the evaluation metric which the higher is better.

## 4. Discussion

Extensive experiments illustrate that our method outperforms the state-of-the-art methods on 8-state secondary structure prediction. Furthermore, we prove the efficiency of our model on boundary residue prediction task. The prediction of boundary residues proposed in this paper provides a new idea for protein sequence study and it also promotes the understanding and application of deep learning for specific tasks of protein. In the future, we will apply our model to more protein related tasks, such as dihedral angles and solvent accessibility.

## 5. Conclusions

In this paper, we propose an ensemble learning encoder with Atrous Spatial Pyramid deep learning model (Ensemble-ASP) for protein secondary structure prediction. The framework contains ensemble learning encoder network and ASP network(modified Atrous Spatial Pyramid Pooling network). Extensive experiments demonstrate that our ensemble encoder has surpassed the state-of-the-art methods on 8-state prediction performance. In addition, boundary residue experiments confirm that our ASP network is able to obtain better prediction performance, and the final results show that the ASP model can further improve the performance of our encoding network. Although there is the possibility of further improvement in hyper-parameters and details, this is a novel attempt for specific protein prediction tasks, which helps the scientists understand the characteristics of proteins. As one of the most important part of our work, our well defined ASP module reveals that specific network design can be helpful for addressing particular bioinformatics problem. Other than developing novel deep learning algorithms, it is equally important to learn and adapt interdisciplinary research ideas from other fields. Our method is expected to be useful for any protein related prediction tasks, such as dihedral angles and solvent accessibility, which is not limited to protein secondary structure prediction.

## Figures and Tables

**Table 1:** The percentage of each secondary structure type and protein number on the training and test dataset.

Label	Cullpdb	CB513	CASP11	CASP12	CASP13	CASP14
H	0.345	0.309	0.305	0.335	0.089	0.118
B	0.01	0.014	0.011	0.011	0.017	0.028
E	0.217	0.213	0.248	0.211	0.175	0.129
G	0.039	0.037	0.035	0.03	0.024	0.045
I	0.0	0.0	0.005	0.004	0.003	0.005
T	0.113	0.118	0.111	0.109	0.2	0.201
S	0.083	0.098	0.085	0.091	0.175	0.155
L	0.193	0.211	0.2	0.209	0.317	0.318
Protein #	5926	513	85	40	10	15

# represents the number of proteins.

**Table 2:** Q8 accuracy of CNN, CondConv, GCNN and CondGCNN on cb513 dataset with different structural settings.

Network	Experts Num	Blocks Num	Dropout Rate	Q8 acc
Conv	-	-	0.0	0.678
CondConv	3	-	0.0	0.680
**CondConv**	3	-	0.2	**0.685**
CondConv	5	-	0.2	0.684
CondConv	8	-	0.2	0.681
GCNN	-	16	0.1	0.696
**GCNN**	-	32	0.1	**0.698**
GCNN	-	64	0.1	0.677
**CondGCNN**	3	32	0.2	**0.702**

Best models and best scores are marked as **bold**.

**Table 3:** The results of before and after inserting the ASP network into the bLSTM network on CB513 datasets.

Network	FC-HS	ASP-NS	Conv1-NS	Q8 acc
bLSTM-FC	128	-	-	0.699
bLSTM-ASP-FC	128	64	-	0.625
**bLSTM-ASP-Conv1**	-	64	100	**0.703**

Best models and best scores are marked as **bold**.

**Table 4:** The results of before and after inserting the ASP network into the ACLSTM network on CB513 datasets.

Network	FC-HS	ASP-NS	Conv1-NS	Q8 acc
ACLSTM-FC	128	-	-	0.705
ACLSTM-ASP-FC	128	64	-	0.706
**ACLSTM-ASP-Conv1**	-	64	100	**0.711**

Best models and best scores are marked as **bold**.

**Table 5:** Hyper-parameter space and best values.

Hyper-Parameter	Values	Best
CondGCNN blocks num	16, 32, 64	32
CondGCNN node size	32, 64, 128	64
CondGCNN experts num	3, 5, 8	3
bLSTM stacked layer	1, 2, 3	2
bLSTM hidden size	256, 512, 1024	512
Output layer	fc, conv1	conv1
Initial learning rate	0.01, 0.001, 0.0001	0.001
Dropout rate	0.0, 0.1, 0.2, 0.3, 0.4	0.2

**Table 6:** The comparison between the Q8 results (the mean and standard deviation measured over the proteins within each test set) of our method and the results of state-of-the-art methods.

Methods	CB513	CASP11	CASP12	CASP13	CASP14
ICML2014	0.664	-	-	-	-
DeepCNF *	0.683 ± 0.128	0.707 ± 0.105	0.681 ± 0.117	0.639 ± 0.118	0.527 ± 0.114
BLSTM *	0.699 ± 0.123	0.711 ± 0.097	0.681 ± 0.118	0.646 ± 0.117	0.556 ± 0.121
CBRNN	0.702	-	-	-	-
DeepACLSTM *	0.705 ± 0.133	0.715 ± 0.099	0.678 ± 0.121	0.647 ± 0.119	0.551 ± 0.148
MUFOLD-SS *	0.704 ± 0.135	0.717 ± 0.107	0.684 ± 0.114	0.651 ± 0.128	0.558 ± 0.130
Ensemble (ours)	0.717 ± 0.131	0.721 ± 0.097	0.686 ± 0.114	0.652 ± 0.107	0.567 ± 0.114
**Ensemble-ASP (ours)**	**0.719 ± 0.135**	**0.728 ± 0.096**	**0.691 ± 0.115**	**0.664 ± 0.104**	**0.572 ± 0.112**

* Data is generated by our experiment. Best model and best scores are marked as **bold**.

**Table 7:** The comparison between the Q8 results on SPOT-1D benchmark dataset (the mean and standard deviation measured over the proteins within each test set) of our method and the results of state-of-the-art methods.

Methods	test2016	test2018
DeepCNF *	0.717 ± 0.099	0.707 ± 0.129
BLSTM *	0.719 ± 0.099	0.712 ± 0.114
MUFOLD-SS *	0.726 ± 0.100	0.710 ± 0.133
DeepACLSTM *	0.729 ± 0.098	0.717 ± 0.121
**Ensemble-ASP**	**0.734 ± 0.098**	**0.723 ± 0.128**

* Data is generated by SPOT-1D benchmark. Best model and best scores are marked as **bold**.

**Table 8:** The boundary residue Q8 accuracy of before and after using the ASP network for the bLSTM, ACLSTM and ensemble (ours) network on CB513 datasets.

Network	Without ASP Module	With ASP Module
bLSTM	0.543	**0.581**
ACLSTM	0.563	**0.591**
Ensemble (ours)	0.582	**0.604**

Best scores are marked as **bold**.

## Data Availability

Input feature extraction during this work and the network structure source codes can be available online at https://github.com/yuzhiguo07/Protein-EnsembleASP.
